# ﻿Leaf epidermal micromorphology of *Zingiber* (Zingiberaceae) from China and its systematic significance

**DOI:** 10.3897/phytokeys.190.77526

**Published:** 2022-02-28

**Authors:** Hui Zhao1, Mei-Hua Xiao*, Yan Zhong, Ying-Qiang Wang

**Affiliations:** 1 Guangdong Provincial Key Laboratory of Biotechnology for Plant Development, School of Life Sciences, South China Normal University, Guangzhou 510631, China South China Normal University Guangzhou China; 2 Guangzhou Key Laboratory of Subtropical Biodiversity and Biomonitoring, School of Life Sciences, South China Normal University, Guangzhou 510631, China South China Normal University Guangzhou China

**Keywords:** crystal, oil cell, stomata, trichome, Zingiberales

## Abstract

Leaf epidermal characteristics are important for phylogenetic and taxonomic studies of many plants, but there is currently insufficient such data for this application in *Zingiber* species. Therefore, the leaf epidermal micromorphology of 22 species in three sections of *Zingiber* was investigated by light microscopy and scanning electron microscopy. Differences between various taxonomic groups of Zingiberaceae were also compared to assess their phylogenetic and taxonomic significance. As in other genera of Zingiberaceae, the epidermal cells in both the adaxial and abaxial epidermis of *Zingiber* species were found to be hexagonal or polygonal, with non-sinuous anticlinal walls that are arranged parallel to leaf veins. Tetracytic stomata are mostly randomly distributed in the intercostal regions of both surfaces and are more common on the abaxial surface. The stomatal density of the species in sect. Pleuranthesis is significantly lower than that in sects. *Zingiber* and *Cryptanthium*. There are two types of trichome in *Zingiber*: so-called “delicate” trichomes are present in most species, while “stout” trichomes with a swollen base are only found in *Z.corallinum* and *Z.montanum*. Oil cells occur in both epidermal layers of some species in sects. *Zingiber* and *Cryptanthium*, but only in the abaxial epidermis of *Z.ellipticum* in sect. Pleuranthesis. Crystals are found in the abaxial epidermis only in all species, but are present in both epidermal layers of *Z.corallinum* and *Z.montanum*. Although the epidermal morphology is similar in most *Zingiber* species, stomatal density, type of trichome and distribution of oil cells and crystals offer valuable information for the systematic and taxonomic studies in this genus.

## ﻿Introduction

The type genus *Zingiber* of Zingiberaceae was established by Miller in 1754 and contains about 150 species, widely distributed from tropical to subtropical Asia (Theilade 1999; Theerakulpisut et al. 2012). The center of *Zingiber* diversity is located in Southeast Asia, where China has 42 species (Wu and Larsen 2000). Given the recent discovery of several new species, this figure is probably an underestimate (Bai et al. 2015a, 2016, 2018; Li et al. 2020a; Wang et al. 2020). *Zingiber* has been confirmed to be monophyletic by molecular analyses (Kress et al. 2002; Li et al. 2020b) and is easily distinguished from other genera of Zingiberaceae by specific features of the flower structure; for example, the lateral staminodes are fused to the labellum; there is an elongated, horn-shaped anther crest wrapped around the upper part of style; and there is a pulvinus at the base of the petiole (Wu and Larsen 2000; Bai et al. 2015b). The traditional infrageneric classification of *Zingiber* recognizes four sections based on the habit and position of the inflorescence (Schumann 1904), namely sects. *Zingiber* (basal with long peduncle), *Cryptanthium* (radical with short procumbent peduncle), *Pleuranthesis* (spikes breaking through the leaf sheaths laterally) and *Dymczewiczia* (terminal inflorescence). However, some species (such as *Z.junceum* and *Z.barbatum*) have been found to have both basal and terminal inflorescences, making it difficult to place them unequivocally in a particular section (Kishor and Leong-Škorničková 2013). A recent study proposed that sect. Dymczewiczia should be merged with sect. Zingiber because palynological evidence suggests that the pollen is very similar in both sections, being spherical with cerebroid sculpturing, which differs from the ellipsoidal pollen grains with spira-striate sculpturing found in sect.Cryptanthium (Theilade et al. 1993). Phylogenetic evidence also shows that sect. Dymczewiczia is nested within sect.Zingiber in the phylogenetic tree (Theerakulpisut et al. 2012). In addition, the taxonomic identiﬁcation of this genus tends to rely on floral characters, mainly flower color and labellum shape (Wu and Larsen 2000), while leaf features have received little attention. However, the flower characters are ineffective in identifying related species due to variation within the same species (Bai et al. 2015b). For example, *Z.monglaense* and *Z.flavomaculosum* have the same leaf and flower characteristics, and are considered to be merged (Tong 1998). The same was observed for *Z.nudicarpum*, *Z.peninsulare* and *Z.newmanii* (Bai et al. 2019). Moreover, the flower characters can be difficult to define precisely in *Zingiber* species, because the florescence time is short and the characters are often not well preserved in herbarium specimens (Bai et al. 2015b). Thus, more experimental studies, for example, involving leaf characteristics and leaf epidermal micromorphology, are needed on the infrageneric systematic classification of *Zingiber*. Leaves have some advantages over flowers, as they can be obtained at all stages of plant growth and leaf features are easier to preserve.

Leaf epidermal micromorphology, which describes the shape of epidermal cells, the outline of anticlinal walls, stomatal type, surface ornamentation and trichome type, has become a tool for the study of phylogeny and taxonomy in many plant species (Baranova 1972; Wilkinson 1979; Stace 1984), especially in those families where identification is complicated, such as Salicaceae (Chen et al. 2008; Ghahremaninejad et al. 2012; Wang et al. 2012), Rosaceae (Tahir and Rajput 2009; Zamani et al. 2017) and Lamiaceae (Moon et al. 2009, 2010; Eiji and Salmaki 2016; Mannethody and Purayidathkandy 2018; Gul et al. 2019). For example, papilla patterns were useful in the discrimination of several Poaceae species (Zhang et al. 2014). The type of trichome, and the form of the trichome base and anticlinal cell walls have all been valuable for the identiﬁcation of Fagaceae species (Zhou and Xia 2012; Deng et al. 2013, 2014). The trichome type and stomatal length were instrumental in assessing the phylogeny of the Ranunculaceae (Hoot 1991; Shi and Li 2003). Based on evidence that the range of variation in leaf epidermis overlaps completely between the two families, den Hartog and Baas (1978) transferred Hippocrateaceae into Celastraceae. In Zingiberaceae, leaf anatomical characters have also proved useful for taxonomic studies. The subfamily Costoideae (= Costaceae) is anatomically very distinct from the remainder of the family, and thus supports the separation of Costoideae from the Zingiberaceae (Tomlinson 1956). The type of silica inclusion serves to distinguish the tribes Globbeae, Hedychieae and Alpinieae (Tomlinson 1956).

The leaf epidermal features of some genera of Zingiberaceae, such as *Amomum*, *Alpinia*, *Boesenbergia*, *Kaempferia*, *CurcumaHedychium*, *Elettaria* and *Globba*, have been described to some extent (Tomlinson 1956; Patel 1975; Hussin et al. 2000, 2001; Xiao et al. 2004; Talip et al. 2005; Jayasree 2007; Chen and Xia 2010; Martins et al. 2010; Tang et al. 2010; Kajornjit et al. 2018; Salasiah and Meekiong 2018). Nevertheless, only a few representative species in these genera have been studied. So far, studies of leaf epidermis of *Zingiber* have only covered seven species (Olatunji 1980; Nyawuame and Gill 1990; Jayasree 2007), including two widely cultivated species (*Z.officinale* and *Z.montanum*), one widely distributed species (*Z.zerumbet*), and four taxa from South India, but these accounts were not detailed. All seven species studied are from two sections, sects. *Zingiber* and *Cryptanthium* (only one species, *Z.wightianum*), which is insufficient to represent the entire genus. In this study, we used multiple samples from the three sections of *Zingiber* (sects. *Zingiber*, *Cryptanthium* and *Pleuranthesis*) in China to investigate leaf epidermal micromorphology by light microscopy (LM) and scanning electron microscopy (SEM), and then compared the leaf epidermal characters at different classification levels in Zingiberaceae. Thus, the aim of the present study was to describe the leaf epidermal features of *Zingiber* and to assess their phylogenetic and taxonomic significance.

## ﻿Materials and methods

More than 300 samples from 22 *Zingiber* species (Table 1) in China were used in the study. Leaf material from mature plants was collected by the authors in the ﬁeld and voucher specimens were deposited in the herbarium of South China Normal University (SN). Fresh leaves were fixed in 90% ethyl alcohol solution, 5% formaldehyde and 5% acetic acid at a ratio of 18:1:1, and subsequently epidermal tissue was obtained from the leaves by gently scraping it off with a stainless steel blade. Pieces of leaf epidermis were stained in a solution of 1% safranin in 50% ethanol, and then dehydrated in an ethanol series before being mounted in glycerine gel for light microscopy. To ensure consistency of epidermal structure, at least five slides were examined for each sample. Twenty epidermal cells and stomata from each sample were measured and a mean was calculated based on the range of variation. The stomatal index and stomatal density were obtained for an area of 0.5 mm × 0.6 mm using the following formulae: stomatal index = number of stomatal apparatuses/ (number of stomatal apparatuses + number of epidermal cells); stomatal density = number of stomatal apparatuses per mm^2^ leaf area. All statistical analyses were performed using SPSS11.5 and Microsoft Excel 2010. A conﬁdence level of *p* ≤ 0.05 was considered to be signiﬁcant. Material for scanning electron microscopy was macerated in 4% glutaric dialdehyde solution for about 24 h and dehydrated in a graded alcohol series, and then mounted on stubs. After gold sputtering, the specimens were examined and photographed under a JEOL JSM-6360LV scanning electron microscope. The terminologies of the stomatal complex types used in this study are those of Fryns-Claessens and Van Cotthem (1973) and Dilcher (1974).

**Table 1.** Comparable leaf epidermal characters of 22 *Zingiber* species. Numbers indicate mean ± standard deviation. Stomatal index = number of stomatal apparatuses/ (number of stomatal apparatuses + number of epidermal cells); Stomatal density = number of stomatal apparatuses/ mm^2^ leaf area.

Taxa	Voucher	Adaxial epidermis				Abaxial epidermis			
		Epidermal cell size (L ×W) (μm)	Stomatal size (L ×W) (μm)	Stomatal index	Stomatal density (mm^-2^)	Epidermal cell size (L ×W) (μm)	Stomatal size (L ×W) (μm)	Stomatal index	Stomatal density (mm^-2^)
Sect. Pleuranthesis									
*Z.ellipticum*	xmh-14-23	72.46±13.15 × 37.04±5.62	37.42±3.30 × 27.57±3.28	0.16±0.26	0.28±0.44	61.38±8.43 × 46.78±6.80	36.73±2.25 × 27.56±1.92	3.67±0.95	19.30±4.94
Sect. Zingiber		64.85(53.41–76.52) × 37.01(30.54–46.59)	43.02(37.99–47.45) × 26.18(21.42–30.37)	1.53(0.22–2.87)	8.08(1.06–19.05)	49.64(40.98–56.47) × 34.98(28.65–43.45)	40.64(36.14–46.90) × 23.69(19.86–26.45)	6.70(5.12–9.17)	60.92(45.93–79.20)
*Z.corallinum*	wyq-14-46	75.27±10.41 × 46.59±6.79	47.45±2.20 × 26.57±2.21	1.54±0.69	3.93±2.38	56.47±9.05 × 43.45±6.40	46.90±3.77 × 24.26±1.69	5.67±0.79	45.93±6.89
*Z.neotruncatum*	xmh-15-16	76.52±12.36 × 30.54±4.04	44.37±3.49 × 30.37±2.43	0.22±0.16	1.06±0.78	55.46±10.12 × 28.65±4.15	38.42±1.99 × 25.29±1.86	5.12±0.69	48.60±6.99
*Z.nudicarpum*	wyq-14-22	57.44±14.90 × 34.15±2.68	37.99±1.46 × 25.20±1.30	0.41±0.28	2.33±2.06	46.25±4.82 × 32.56±2.15	36.14±2.86 × 22.59±4.54	6.82±0.59	67.74±10.38
*Z.montanum*	wyq-15-65	61.63±3.08 × 42.83±3.64	43.97±3.37 × 27.32±1.36	2.87±0.90	14.02±3.73	49.04±2.38 × 38.62±1.25	45.55±3.37 × 26.45±1.16	9.17±1.69	63.11±6.36
*Z.zerumbet*	wyq-14-44	53.41±1.74 × 30.96±1.83	41.32±2.60 × 21.42±0.38	2.60±0.51	19.05±2.31	40.98±2.56 × 31.61±2.73	36.20±0.52 × 19.86±0.21	6.70±0.52	79.20±3.98
Sect. Cryptanthium		73.49(61.10–86.03) × 36.71(28.81–45.61)	42.97(37.08–54.83) × 27.20(23.08–32.07)	1.12(0.45–2.19)	5.48(1.99–14.52)	55.12(43.36–72.89) × 39.35(31.06–49.64)	41.81(34.78–49.11) × 25.00(21.41–29.15)	6.80(4.74–9.34)	49.55(23.59–88.46)
*Z.atrorubens*	hn-zzj-14-01	72.44±15.14 × 39.86±7.24	54.83±3.47 × 28.82±2.33	2.19±0.68	7.98±2.41	55.35±5.73 × 44.39±6.19	44.50±4.22 × 26.21±2.37	6.32±1.66	28.49±11.54
*Z.bisectum*	xmh-14-15	62.18±8.02 × 37.53±2.19	44.35±2.14 × 26.51±5.54	1.39±0.35	7.50±1.82	43.36±3.23 × 33.39±1.45	42.16±3.90 × 21.58±3.92	9.34±0.58	88.46±16.44
*Z.cochleariforme*	wyq-14-56	84.36±8.46 × 36.22±2.28	42.45±2.29 × 27.69±3.50	0.55±0.34	1.99±1.73	60.29±5.44 × 41.08±1.97	42.55±3.44 × 25.16±3.42	6.89±2.33	45.27±9.26
*Z.densissimum*	wyq-14-96	84.81±6.75 × 45.15±5.65	44.30±1.98 × 32.07±3.36	0.71±0.25	2.68±1.11	55.74±10.41 × 40.61±5.42	44.52±1.54 × 29.15±2.18	7.33±1.11	50.55±7.22
*Z.flavomaculosum*	wyq-14-72	71.32±9.11 × 35.66±3.76	40.10±4.11 × 25.14±3.20	1.77±0.58	10.29±4.34	52.02±8.43 × 35.21±3.02	37.67±3.14 × 22.79±2.40	7.61±1.07	66.60±14.10
*Z.guangxiense*	wyq-15-10	85.90±7.73 × 38.59±5.70	39.92±2.68 × 27.03±2.21	0.74±0.16	3.33±0.67	60.91±4.95 × 41.88±4.29	40.84±1.73 × 24.30±2.96	7.46±0.96	48.02±8.28
*Z.leptorrhizum*	wyq-15-80	75.27±20.48 × 38.19±11.66	47.38±2.16 × 29.21±3.04	1.31±0.29	4.01±0.87	72.89±9.25 × 49.64±13.12	49.11±1.80 × 27.23±1.66	6.34±1.28	23.59±5.00
*Z.lingyunense*	xmh-14-14	61.71±11.56 × 30.16±5.37	39.46±2.42 × 24.82±2.88	0.45±0.11	3.10±0.78	50.21±7.97 × 37.51±4.43	38.79±1.56 × 23.29±1.38	4.74±0.96	35.93±5.56
*Z.longiglande*	wyq-15-02	67.45±6.28 × 38.92±1.11	41.35±0.61 × 30.47±4.23	0.61±0.03	2.50±0.39	55.86±5.17 × 39.22±5.42	43.12±4.13 × 27.90±0.65	7.08±0.08	45.45±3.72
*Z.longiligulatum*	wyq-14-124	71.47±6.28 × 37.80±5.31	45.26±5.28 × 28.84±3.07	1.10±0.39	4.73±2.70	54.93±4.42 × 41.40±3.86	44.54±5.53 × 24.48±3.01	7.28±0.77	53.99±7.36
*Z.orbiculatum*	wyq-14-62	61.10±7.42 × 28.81±6.94	37.08±1.09 × 23.08±2.05	0.94±0.68	14.52±3.36	47.97±6.74 × 31.06±4.77	34.78±0.80 × 21.41±1.89	6.25±0.63	45.97±19.12
*Z.recurvatum*	wyq-14-80	72.28±7.50 × 33.14±2.82	38.54±2.65 × 27.13±3.65	0.87±1.12	4.76±6.57	53.68±6.15 × 36.29±5.00	38.16±1.61 × 23.50±2.21	7.22±1.26	56.01±5.61
*Z.roseum*	wyq-14-122	86.03±9.60 × 45.61±6.54	42.86±2.98 × 30.08±2.16	0.83±0.24	2.67±0.97	63.33±17.15 × 40.86±5.19	42.68±2.79 × 28.56±1.85	7.42±0.70	53.46±5.91
*Z.teres*	wyq-14-119	75.28±20.29 × 31.27±2.97	38.07±1.26 × 25.27±5.41	0.85±0.59	5.43±4.35	46.99±3.42 × 35.27±1.50	37.43±3.27 × 24.03±1.79	6.47±0.23	68.04±6.42
*Z.tuanjuum*	wyq-14-54	80.88±9.37 × 41.32±5.30	49.75±3.23 × 25.63±2.16	1.03±0.17	4.90±0.76	57.07±7.61 × 45.28±6.95	47.05±2.78 × 27.87±1.93	5.86±0.73	37.56±6.19
*Z.xishuangbannaense*	wyq-14-87	63.40±7.31 × 29.04±2.04	41.86±2.85 × 23.45±0.83	1.52±0.43	7.22±6.22	51.29±3.44 × 36.54±1.21	41.05±2.52 × 22.50±3.66	5.35±0.28	45.45±11.68

## ﻿Results

A comparison of leaf epidermal characteristics in 22 *Zingiber* species is shown in Table 1.

### ﻿Epidermal cells

**Figure 1.**
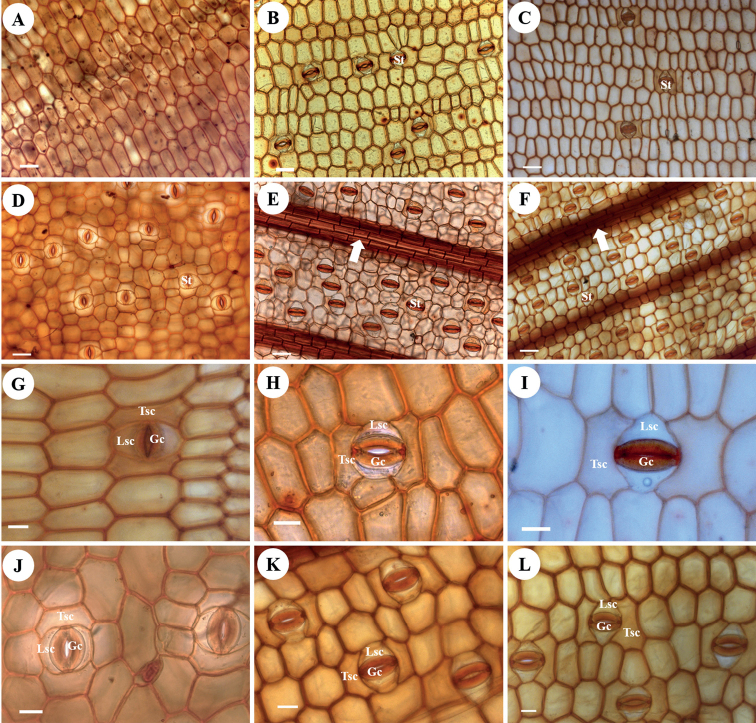
Leaf epidermal characters of *Zingiber* shown by light microscopy **A–C** adaxial epidermis of *Z.ellipticum* (**A**), *Z.montanum* (**B**) and *Z.flavomaculosum* (**C**) showing epidermal cells and stomatal apparatus **D–F** abaxial epidermis of *Z.ellipticum* (**D**), *Z.montanum* (**E**) and *Z.teres* (**F**) showing epidermal cells, costal epidermal cells and stomatal apparatus (arrows indicate to the costal epidermal cells) **G–I** detail of tetracytic stomatal apparatus on the adaxial epidermis of *Z.ellipticum* (**G**), *Z.montanum* (**H**) and *Z.tuanjuum* (**I**) **J–L** detail of tetracytic stomatal apparatus in the abaxial epidermis of *Z.ellipticum* (**J**), *Z.montanum* (**K**) and *Z.longiligulatum* (**L**). St: stoma; Gc: guard cell; Lsc: lateral subsidiary cell; Tsc; terminal subsidiary cell. Scale bars: 50 μm (**A–F**); 20 μm (**G–I**).

**Figure 2.**
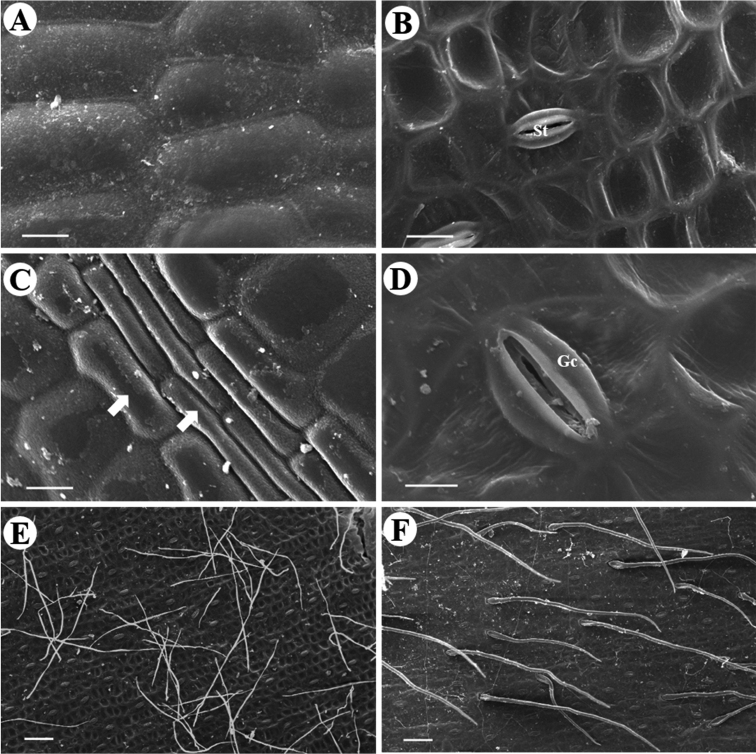
Leaf epidermal characters of *Zingiber* shown by scanning electron microscopy **A** adaxial epidermis of *Z.flavomaculosum* showing convex epidermal cells with smooth cuticular membranes **B** abaxial epidermis of *Z.xishuangbannaense* showing concave epidermal cells with smooth cuticular membranes **C** detail of epidermis over the vein in *Z.montanum* (arrows indicate costal epidermal cells) **D** stomatal apparatus in *Z.flavomaculosum* showing guard cells with smooth cuticular membranes **E** delicate trichomes in *Z.xishuangbannaense***F** stout trichomes with swollen trichome base in *Z.corallinum*. St: stoma; Gc: guard cell. Scale bars: 10 μm (**D**); 20 μm (**A–C**); 100 μm (**E, F**).

When examined by LM, the epidermal cells of *Zingiber* species were found to be mostly hexagonal or polygonal, with the long axis usually perpendicular to the veins, and arranged in rows parallel to the veins; the anticlinal walls were straight to slightly curved (Fig. 1A–F). Adaxial epidermal cells were always more regularly arranged and slightly larger than abaxial epidermal cells (Table 1). Adaxial epidermal cells ranged in size from 53.41±1.74 × 30.96±1.83 um (in *Z.zerumbet*) to 86.03±9.60 × 45.61±6.54 um (in *Z.roseum*), and were usually elongated with the length 1.4–3.2 times longer than the width. The abaxial epidermal cells ranged from 40.98±2.56 × 31.61±2.73 um (in *Z.zerumbet*) to 72.89±9.25 × 49.64±13.12 um (in *Z.leptorrhizum*) in size, and also were usually elongated with the length 1.2–2.0 times longer than the width. The epidermal cells above the veins were smaller and more or less longitudinally elongated (Figs 1E, F, 2C). When examined by SEM, the cells were convex on the adaxial side of the epidermis (Fig. 2A) and concave on the abaxial side (Fig. 2B) with smooth cuticular membranes. The anticlinal cell walls were invisible or obscure.

### ﻿Stomatal apparatus

The stomatal apparatus, which occurs in both the adaxial and abaxial leaf epidermis in all *Zingiber* species studied, was of the tetracytic type with four subsidiary cells around the stoma, one on each side and one at each pole (Fig. 1G–L). The guard cells were reniform with smooth cuticular membranes (Fig. 2D). The lateral subsidiary cells were subtriangular with the long axis parallel to the stoma, while the terminal subsidiary cells were adjacent to the stoma poles. The stomatal orientation (the long axis of all stomata) was approximately parallel to the veins. The stomatal size in both leaf epidermal layers was similar in all species (Table 1), but ranged from 41.32±2.60 × 21.42±0.38 um (in *Z.zerumbet*) to 54.83±3.47 × 28.82±2.33 um (in *Z.atrorubens*) in the adaxial epidermis and from 36.20±0.52 × 19.86±0.21 um (in *Z.zerumbet*) to 49.11±1.80 × 27.23±1.66 um (in *Z.leptorrhizum*) in the abaxial epidermis. Stomata occurred much more frequently in the abaxial epidermis than in the adaxial epidermis in all species studied (Table 1), and most of them were randomly distributed in the intercostal regions (Fig. 1E, F). The stomatal index of the adaxial epidermis and the abaxial epidermis ranged from 0.16%±0.26% (in *Z.ellipticum*) to 2.87%±0.90% (in *Z.montanum*) and from 3.67%±0.95% (in *Z.ellipticum*) to 9.34%±0.58% (in *Z.bisectum*), respectively. The stomatal density of the adaxial epidermis and the abaxial epidermis ranged from 0.28±0.44 (in *Z.ellipticum*) to 19.05±2.31 (in *Z.zerumbet*) and from 19.30±4.94 (in *Z.ellipticum*) to 88.46±16.44 (in *Z.bisectum*), respectively.

### ﻿Trichomes

**Figure 3.**
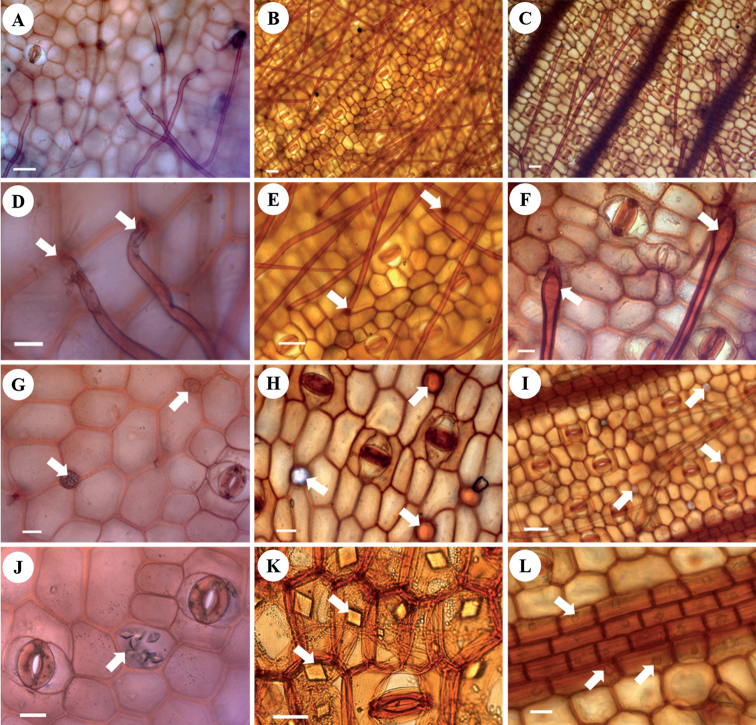
Characters of trichomes, oil cells and crystals in leaf epidermis of *Zingiber* shown by light microscopy **A, B** delicate trichome of *Z.ellipticum* (**A**) and *Z.densissimum* (**B**) **C** stout trichome of *Z.corallinum***D, E** detail of delicate trichome of *Z.ellipticum* (**D**) and *Z.densissimum* (**E**) showing the trichome base (white arrows) **F** detail of stout trichome of *Z.corallinum* showing the swollen trichome base (white arrows) **G–I** oil cells (white arrows) of *Z.ellipticum* (**G**), *Z.orbiculatum* (**H**) and *Z.montanum* (**I**) **J, K** crystals distributed in the epidermal cells (white arrows) of *Z.ellipticum* (**J**) and *Z.guangxiense* (**K**) **L** crystals distributed above the veins (arrow pointing to crystal) of *Z.montanum*. Scale bars: 50 μm (**A, B, C, E, I, K**); 20 μm (**D, F, G, H, J, L**).

Trichomes were found on the abaxial surface in all species studied (Figs 2E, F, 3A–C), and occasionally also on the adaxial surface in *Z.ellipticum*, *Z.xishuangbannaense* and *Z.bisectum*. Two types of trichome were recorded: Type 1, a delicate, simple unicellular trichome, straight or curly, and easily detached (Figs 2E, 3A, B, D, E), which was found in all species studied except for *Z.corallinum* and *Z.montanum*; Type 2, a stout, simple unicellular trichome, straight with pointed apex and swollen trichome base (Figs 2F, 3C, F), which was found in only two species, *Z.corallinum* and *Z.montanum*.

### ﻿Oil cells

Oil cells occurred frequently on the abaxial surface in all samples of the *Zingiber* species studied, and also occurred infrequently in the adaxial epidermis of *Z.densissimum*, *Z.longiligulatum*, *Z.roseum* and *Z.xishuangbannaense*. The oil cells were subrotund and of small size, and usually contained yellow or translucent oil droplets (Fig. 3G–I), which were easily distinguishable from epidermal parenchyma cells.

### ﻿Crystals

Crystals were found in the epidermis of all *Zingiber* species studied. There were many crystals in the epidermis of *Z.corallinum*, *Z.montanum*, *Z.longiglande*, *Z.tuanjuum*, *Z.cochleariforme*, *Z.guangxiense* and *Z.teres*, but few in *Z.atrorubens*, *Z.recurvatum* and *Z.leptorrhizum*; crystals were rare in the remaining species. The crystals were usually rhombic and square (Fig. 3J–L), and were mainly distributed above or near the veins in the abaxial epidermis (Fig. 3L), as well as in the intercostal regions (Fig. 3J, K), while only being present in the adaxial epidermis of *Z.corallinum* and *Z.montanum*.

## ﻿Discussion

Our results show that the epidermal cells of *Zingiber* species are very similar in shape, i.e. hexagonal or polygonal, with non-sinuous anticlinal walls; the cells are arranged parallel to leaf veins. The results are consistent with those of previous studies on seven species of *Zingiber* (Olatunji 1980; Nyawuame and Gill 1990; Jayasree 2007) and other genus species in Zingiberaceae (Table 2).

**Table 2.** Comparable leaf epidermal characters of the nine genera in Zingiberaceae.

Genus	Epidermal cell		Stomatal apparatus		Trichome	Oil cell	References
	Shape	Anticlinal wall	Type	Distribution			
*Boesenbergia*	hexagonal or polygonal	not sinuous	tetracytic	randomly distributed in the intercostal regions	delicate trichome	present in abaxial epidermis	d, e
*Curcuma*	polygonal	not sinuous	tetracytic	distributed in the intercostal regions	stout trichome	present in abaxial epidermis	b, e, k, m, l
*Hedychium*	polygonal	not sinuous	tetracytic	distributed in the intercostal regions, sometimes above the veins	delicate trichome	present in abaxial epidermis	e, g, l
*Kaempferia*	polygonal	not sinuous	tetracytic	distributed in the intercostal regions	stout trichome and delicate trichome	present in both epidermal layers	d, e, l
*Globba*	polygonal	not sinuous	tetracytic	randomly distributed in the intercostal regions or distributed in rows near veins	delicate trichome	present in both epidermal layers	e, f, l
*Zingiber*	hexagonal or polygonal	not sinuous	tetracytic	randomly distributed in the intercostal regions	stout trichome and delicate trichome	frequently present in the abaxial epidermis; also occurs in the adaxial epidermis	a, e, l
*Alpinia*	polygonal	not sinuous	tetracytic	randomly distributed in the intercostal regions or distributed in rows near veins	stout trichome	present in abaxial epidermis	c, e, i, j, l
*Amomum*	polygonal	not sinuous	tetracytic	distributed in the intercostal regions	stout trichome	present in abaxial epidermis	e, i, k, l
*Elettaria*	hexagonal or polygonal	not sinuous	tetracytic	more frequent distributed closer to the veins	stout trichome	present in abaxial epidermis	e, i, l

Notes: a, this study; b, Chen and Xia 2010; c, Hussin et al. 2000; d, Hussin et al. 2001; e, Jayasree 2007; f, Kajornjit et al. 2018; g, Martins et al. 2010; h, Patel 1975; i, Salasiah and Meekiong 2018; j, Talip et al. 2005; k, Tang et al. 2010; l, Tomlinson 1956; m, Xiao et al. 2004.

Similarly to a number of other Zingiberaceae genera, the stomata of *Zingiber* are amphistomatic, tetracytic and aligned in a linear-axial orientation (Table 2). The distribution of the stomatal apparatus can be useful for taxonomic studies of Zingiberaceae. For example, the stomatal apparatus of three genera, *Alpinia*, *Elettaria* and *Globba*, are distributed near the veins in rows, as well as being randomly distributed in the intercostal regions, while in most other genera of Zingiberaceae the stomata are always randomly distributed in the intercostal regions. These three genera can therefore be distinguished on this basis from other genera of Zingiberaceae. In addition, our results show that the stomatal density and stomatal index of the species in sect. Pleuranthesis are significantly lower than in sects. *Zingiber* and *Cryptanthium*. This suggests that stomatal density (or stomatal index) could allow species of sect. Pleuranthesis to be distinguished from other species of *Zingiber*.

Previous studies (Tomlinson 1956) found two types of trichome on the epidermis of Zingiberaceae, the stout trichome (“Borste”) and the delicate trichome (“Weichhaare”). Our results also show these two types of trichomes on the epidermis of the *Zingiber* species studied. Similarly to other genera of Zingiberaceae, all trichomes on the leaf surfaces of *Zingiber* were unicellular (Table 2). However, delicate trichomes were found on the epidermis of most species of *Zingiber*, while stout trichomes (“Borste”) were only found in two species, *Z.corallinum* and *Z.montanum*. This indicates that *Z.corallinum* and *Z.montanum* are closely related and markedly different from other species of *Zingiber*. In addition, the trichomes on the epidermis of three genera (*Alpinia*, *Amomum* and *Elettaria*) from the subfamily Alpinioideae are all “Borste” (Table 2), while those of the genera in the subfamily Zingiberoideae are either “Borste” and/or “Weichhaare”. This indicates that the type of trichome can have taxonomic significance in Zingiberaceae. According to various molecular phylogenetic trees (Kress et al. 2002, Williams et al. 2004, Liang et al. 2020), the stout trichome exists in both the derived groups (such as *Kaempferia*, *Hedychium* and *Zingiber*) and basal groups (such as *Aplinia*, *Amomum* and *Elettaria*) of Zingiberaceae, while the delicate trichome is only present in the derived groups. Within the tribe Zingibereae, the genus *Curcuma* only has the stout trichome, while the other three genera, *Kaempferia*, *Hedychium* and *Zingiber*, have both types. This suggests that the latter three genera should be closely related to each other, and more distantly related to *Curcuma*, consistent with the molecular phylogenetic trees (Kress et al. 2002; Liang et al. 2020). These results also suggest that the characteristics of leaf epidermal trichomes have systematic and taxonomic significance for Zingiberaceae.

Previous studies have shown that oil cells, which often occur in the mesophyll, root and rhizome (Sherlija et al. 1998, Tang et al. 2010, Uma and Muthukumar 2014), are present in all species of Zingiberaceae (Tomlinson 1959). Oil cells are responsible for the production of volatile compounds that provide fragrance for the leaves of Zingiberaceae species (Victório et al. 2011). We found that oil cells occur in the leaf abaxial epidermis of all *Zingiber* species, and also occur in the adaxial epidermis of three species from sect. Cryptanthium, *Z.longiligulatum*, *Z.roseum* and *Z.densissimum*, and one species from sect. Zingiber, *Z.xishuangbannaense*, but not at all in the leaf adaxial epidermis of the primitive sect. Pleuranthesis. Similarly, oil cells in most genera of Zingiberaceae are found mainly in the abaxial epidermis, but in the adaxial epidermis of only three genera of the subfamily Zingiberoideae (Table 2), *Kaempferia*, *Globba* and *Zingiber*. Thus, the distribution of oil cells is a useful characteristic in identifying species and sections of *Zingiber*, as well as genera of Zingiberaceae.

Crystals are usually rhombohedral, rod-like or acicular, sometimes occurring in clusters that resemble a coarse sand, and are commonly found in the hypodermis of the lamina in families of Zingiberales, such as Musaceae, Cannaceae and Heliconiaceae, but rarely in leaf epidermis (Tomlinson 1959, 1961, Triplett and Kirchoff 1991). Crystals were recently found in costal epidermal cells on both leaf surfaces in some genera of Zingiberaceae, such as *Globba*, *Alpinia*, *Amomum* and *Elettaria* (Tomlinson 1956; Hussin et al. 2000; Talip et al. 2005; Jayasree 2007; Kajornjit et al. 2018; Salasiah and Meekiong 2018). We also found crystals in the epidermal cells of all species of *Zingiber*. The crystals only occur in the abaxial epidermis of most species of *Zingiber*, but in both epidermal layers of *Z.corallinum* and *Z.montanum*, showing that the two species *Z.corallinum* and *Z.montanum* are closely related and markedly different from other species of *Zingiber*. Thus, crystals in leaf epidermis can also have systematic and taxonomic significance for *Zingiber*.

*Z.ellipticum*, the sole member of sect. Pleuranthesis in China, was preliminarily identified as a new species, *Plagiostachyselliptica* of the genus *Plagiostachys* by Tong and Xia (1987) based on the character of the spike inflorescence breaking through the leaf sheaths laterally. Subsequently, it was transferred to sect. Pleuranthesis under the genus *Zingiber* by Wu et al. (1996), because of its spherical pollen grains and two floral characters: i) the labellum has basally connate lateral staminodes; ii) the elongated anther appendage is wrapped around the style. The above analyses of leaf epidermal micromorphology of *Zingiber* spp. also show that the leaf epidermal characters of *Z.ellipticum* from sect. Pleuranthesis are basically consistent with other *Zingiber* species, suggesting that this species has a close interrelationship with other *Zingiber* species. However, there are obvious differences in stomatal density in leaf epidermis between *Z.ellipticum* from sect. Pleuranthesis and the species from the other two sections of *Zingiber*. This suggests that the species of sect. Pleuranthesis form a distinct taxon within the genus of *Zingiber*. This has been confirmed by molecular phylogenetics (Kress et al. 2002; Theerakulpisut et al. 2012), but further molecular, morphological, anatomical and palynological studies are needed for a systematic classification. In addition, the two species of sect. Zingiber, *Z.corallinum* and *Z.montanum*, both of which have stout trichomes and crystals in both epidermal layers, are markedly different from other species of *Zingiber*. Moreover, their inflorescence and leaf morphology are similar, which can make their identification confusing. These observations highlight the need for the systematic relationship between and classification of the two species to be determined after further study.

## ﻿Conclusions

As in other genera of Zingiberaceae, the epidermal cells of *Zingiber* are hexagonal or polygonal, with non-sinuous anticlinal walls, with the cells arranged parallel to leaf veins. Tetracytic stomata are distributed on both surfaces, and oil cells and crystals are common. Although the overall epidermal morphology is similar among *Zingiber* species, stomatal density, trichome type and distribution of oil cells and crystals can offer valuable systematic and taxonomic information. Two types of trichome are found in *Zingiber*: delicate trichomes are present in most species, while stout trichomes with a swollen base are present in *Z.corallinum* and *Z.montanum*, which is a novelty for *Zingiber*.
